# Distribution, Genetic Diversity and Biocontrol of Aflatoxigenic *Aspergillus flavus* in Serbian Maize Fields

**DOI:** 10.3390/toxins13100687

**Published:** 2021-09-27

**Authors:** Vanja Vlajkov, Mila Grahovac, Dragana Budakov, Marta Loc, Ivana Pajčin, Dragan Milić, Tihomir Novaković, Jovana Grahovac

**Affiliations:** 1 Faculty of Technology, University of Novi Sad, Bulevar Cara Lazara 1, 21000 Novi Sad, Serbia; ivana.pajcin@uns.ac.rs (I.P.); johana@uns.ac.rs (J.G.); 2 Faculty of Agriculture, University of Novi Sad, Trg Dositeja Obradovića 8, 21000 Novi Sad, Serbia; dragana.budakov@polj.edu.rs (D.B.); marta.loc@polj.edu.rs (M.L.); dragan.milic@polj.edu.rs (D.M.); tihomir.novakovic@polj.uns.ac.rs (T.N.)

**Keywords:** aflatoxin, *Aspergillus flavus*, maize, biocontrol, Bacillus, HPLC, ELISA, biocontrol, Cluster Amplification Patterns analysis

## Abstract

Maize is one of the leading export products in the Republic of Serbia. As a country where economic development depends on agriculture, maize production plays a critical role as a crop of strategic importance. Potential aflatoxin contamination of maize poses a risk to food and feed safety and tremendous economic losses. No aflatoxin contamination of maize samples harvested in 2019 and 2020 in different localities in the Republic of Serbia was detected by the Enzyme-Linked Immunosorbent Assay (ELISA) test and High-Performance Liquid Chromatography (HPLC) method. On the other hand, the Cluster Amplification Patterns (CAP) analyses of the isolated *Aspergillus flavus* strains from 2019 maize samples confirmed the presence of key biosynthesis genes responsible for aflatoxin production. Artificial inoculation and subsequent HPLC analysis of the inoculated maize samples confirmed the high capacity of the *A. flavus* strains for aflatoxin production, pointing to a high risk of contamination under favorable conditions. Prevention of aflatoxin contamination is primarily based on *A. flavus* control, where biocontrol agents play a significant role as sustainable disease management tools. In this study, antagonistic activity screening of the novel strains belonging to the *Bacillus* genus indicated superior suppression of *A. flavus* strains by two *Bacillus* strains isolated from the rhizosphere of *Phaseolus vulgaris.*

## 1. Introduction

The share of agricultural production in the gross domestic product (GDP) in the Republic of Serbia accounts for approximately 10%. The country’s economic development heavily depends on the agricultural sector due to its importance in the food industry, where crops are used as raw materials, and the contribution of agricultural products in international trade. In 2020, maize was ranked second on the list of export products in the Republic of Serbia. It supports the fact that maize is the crop of strategic importance for the country [1]. Speaking globally, there are three leading agricultural crops: wheat, rice, and maize [2]. The maize taking the dominant position in the world agricultural system is explained by the possibility of being used in not only food but other industry branches as a multipurpose crop. High nutritional values (carbohydrates content 70–75%) make maize suitable raw material for food and feed production and define this crop as a critical factor for world nutrition and livelihood security [3].

Numerous fungal plant diseases and their effect on the qualitative and quantitative aspects of food production led to significant economic losses measured in billions of US dollars worldwide [4]. On the other hand, a limited number of fungal pathogens can cause severe problems affecting food safety and profitability of the plant production comparable to the negative impact caused by the *A. flavus* species [5,6]. The estimation of the European Commission (EC) confirms the influence of mycotoxin contamination resulting in annual global crop losses of 5 to 10% [7]. *A. flavus* is an opportunistic fungal pathogen of crops, predominantly maize, peanuts, and cotton, characterized by the high potential for aflatoxin production [5]. Aflatoxins are potent, highly toxic secondary metabolites that can compromise food and feed security and cause severe health issues [8]. Even exposure to low concentrations of aflatoxin increases the risk of immune suppression, malabsorption of nutrients, infertility, and reduction in life expectancy [9,10]. Aflatoxin class considers four major types, including B1, B2, G1, and G2, and from the food safety point of view, the most relevant is aflatoxin B1 (AFB1) [11]. According to the International Agency for Research on Cancer (IARC) AFB1 is characterized as a carcinogen (Group 1a) [11,12].

The aflatoxigenic potential of *A. flavus* varies from atoxigenic to highly toxigenic strains [12]. The incidence of a toxigenic *A. flavus* species has been shown to be associated with geographic origin and substrate characteristics [13]. Previous studies on the distribution of *A. flavus* in maize fields reported a high incidence of atoxigenic strains (above 70%) [14]. The significance of *A. flavus* as a plant pathogen lies in aflatoxin contamination, and less in resulting yield losses as a consequence of plant infection. Additionally, contamination levels and manifested infection symptoms are commonly disproportional. It means that even barely noticeable infection signs could be followed by high-level aflatoxin contamination [11]. Regarding the distribution in the ecological niches, *A. flavus* species are the most common habitants of the tropical environment, with a relatively high temperature range of 28 °C to 37 °C and high relative humidity of about 95% [9]. Under the common climate conditions, the occurrence of the *A. flavus* on the territory of the Republic of Serbia is not typical [11]. However, global climate changes triggered the more frequent occurrence of the *A. flavus* species in the regions generally characterized by the low risk of contamination [15]. In the past decade, several aflatoxin outbreaks worldwide raised awareness about the importance of defining strategies effective in preventing the development of aflatoxigenic strains [16]. As a consequence of the extremely drought 2012 producing season, the Republic of Serbia faced an aflatoxin outbreak in maize fields resulting in significant economic losses [11]. 

The scientific community recognized the application of biocontrol agents as a promising sustainable solution to address the emerging issue [17,18]. Bacteria belonging to the *Bacillus* genus are conceded as a plant beneficial species in agricultural practice due to high antagonistic activity against phytopathogenic organisms, the ability to promote plant growth, and improve the soil quality [19]. The primary role as a biological tool for plant disease management is defined by the outstanding ability of the *Bacillus* species to out-compete the target pathogens by the synergistic activity of antimicrobial compounds, competition for nutrient sources and space, and triggering induction of plant’s defense response [20]. Besides antibiotics, *Bacillus* strains are well known for the production of many other metabolites of interest, including biosurfactants and enzymes [21]. Additional criteria making *Bacillus* species ideal candidates for the application in biocontrol are spore-forming ability, rapid replication, and resistance to adverse environmental conditions [22]. 

Numerous studies have proven the high potential of *Bacillus* species in the suppression of fungal pathogens. Besides promising scientific results and high potential, the full commercialization of microbial biopesticides is still in the preliminary phase, with a limited number of products available on the market. The existing disbalance between the incontestable potential and the current market scenario requires constant research and efforts to isolate novel strains with high antagonistic activity. Estimating their antimicrobial potential and suitability to be used as a central point for designing viable bioprocess solutions is a necessary precondition for boosting the commercialization and broader use of microbial biopesticides [23]. Development of the biotechnological solution for the production of biocontrol agents starts with selecting the strain expressing the highest potential in suppression of the target pathogen. The beginning of the screening procedure typically includes a significant number of potentials producing strains. The rhizosphere soil is a well-known source rich in antagonistic strains. Bacterial strains originating from the rhizosphere naturally coexist in the dynamic environment and constantly interfere with numerous microbial community members [24]. The great advantage of *Bacillus* strains as biocontrol agents is that they are core soil inhabitants, well adapted to the environmental conditions where they should be lately applied in the form of biopesticide [21].

In the Republic of Serbia, an agricultural practice still relies on the usage of chemical pesticides. The biopesticides market in the country is still underdeveloped and accounts for only 1.3% of the overall market of plant protection products. Data on the biopesticides import in the period from 2015 to 2020 indicates that the largest amount of imported biopesticides refers to bioinsecticides, followed by biofungicides, bioacaricides, and biobactericides [25,26]. Currently, there is a limited number of microbial biopesticides registered and available on the Serbian market. To establish the basis for eco-friendly future in plant protection, co-operation between the scientific community, government, and private sector is needed [23].

Since identification and quantification of aflatoxins in food sources are significant steps in food safety management [8], the principal aim of this study was to evaluate the aflatoxigenic potential of *A. flavus* species isolated from the maize originated from the different localities of the Republic of Serbia. Aflatoxin contamination of the maize samples harvested in 2019 and 2020 was evaluated by the HPLC and the ELISA method. The genetic potential of isolated *A. flavus* species in terms of aflatoxin production capacity was examined by CAP analysis. The second aim was to find the effective biological response to the phytopathogenic fungi development by isolating and evaluating the potential of *Bacillus* spp. strains to be used as biocontrol agents, against toxigenic *A. flavus* strains. The novel strains were isolated from the rhizosphere soil of different vegetable plants, originated from localities in the Autonomous Province of Vojvodina, Republic of Serbia. The antagonistic effect of *Bacillus* spp. against toxigenic and atoxigenic *A. flavus* strains was determined by the well diffusion assay.

## 2. Results

### 2.1. Determination of Aflatoxin B1 by the ELISA Test and Total Aflatoxins and Aflatoxin B1 by the HPLC Method

The High-Performance Liquid Chromatography (HPLC) method was applied to determine the aflatoxin B1 (AFB1) and total aflatoxins content in collected samples of maize originated from 10 selected localities harvested in 2019 (Štitar, Valjevo, Pančevo, Sabanta, Subotica, Nadalj, Loznica, Bečej, Sombor, Rogojevac) (Table 1) and 2020 (Rumenka, Oparić, Kuzmin, Lepojević, Martinci, Krušedol, Valjevo, Beška, Bečej, Sombor) (Table 2). Additionally, the aflatoxin B1 content in the maize samples was determined by the Enzyme-Linked Immunosorbent Assay (ELISA) test (Table 3 and Table 4). The obtained results of the ELISA method pointed out that only one sample (LO, 2019) from the Loznica locality has tested positive for the presence of aflatoxin B1 (0.0046 mg/kg). The other samples (95% of the total number) were not contaminated by aflatoxin B1. The results of the HPLC method confirmed the ELISA testing outcome since only the sample originated from Loznica locality (LO) showed a positive result of aflatoxin contamination (0.002 mg/kg). The aflatoxins (including aflatoxin B1) were detected in 1 out of 20 samples (5%).

### 2.2. Macro and Micromorphological Characterization of A. flavus 

**Figure A1:**
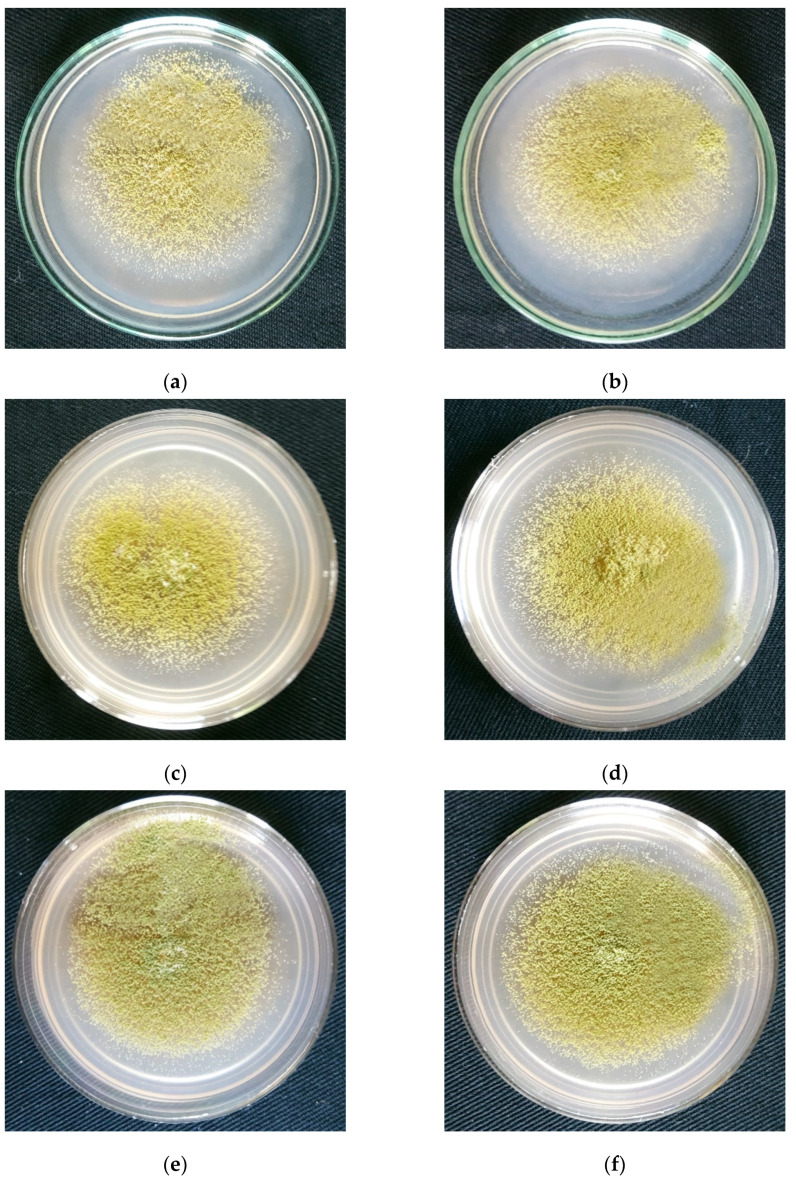

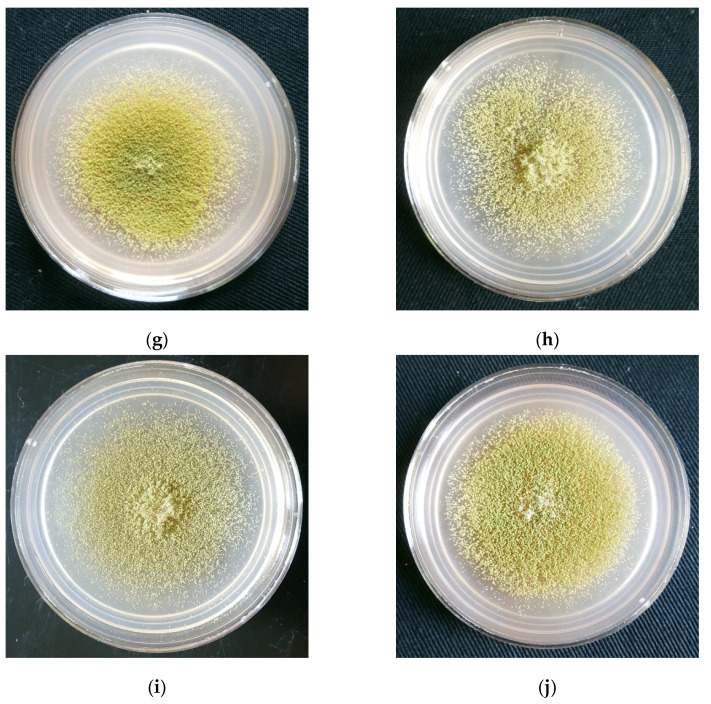
Macromorhological characteristics of *A. flavus* strains. (**a**)—ŠT2BSS, (**b**)—VA1BSS, (**c**)—PA2DSS, (**d**)—SA2BSS, (**e**)—SU1ASS, (**f**)—NA2BSS, (**g**)—LO1ASS, (**h**)—BČ1CSS, (**i**)—SO1ASS, (**j**)—RO2BSS.

Identification of *Aspergillus* spp. single-spore strains isolated from the collected maize samples from ten localities (Štitar, Valjevo, Pančevo, Sabanta, Subotica, Nadalj, Loznica, Bečej, Sombor, Rogojevac) in 2019 to the species level considered macro and micromorphological characterization of the strains, after isolation using the selective medium for *A. flavus* [27]. The results of macroscopic observations of the *Aspergillus* strains are presented in Figure A1 (Appendix C). Initially, the mycelia of *A. flavus* strains were white. After three days of incubation, the colour changes were observed when the sporulation started and progressed radially over the colonies. White soft velvety colonies turned into the yellow-green compact powdery mass with a whitish margin by the end of five days of incubation. The colonies were flat at the borders and raised in the middle. The 5-days old colony diameter ranged from 3.5 cm to 4 cm. The strains also produced exudates that were brown or colourless, while the reverse of the colonies was pale. 

**Figure A2:**
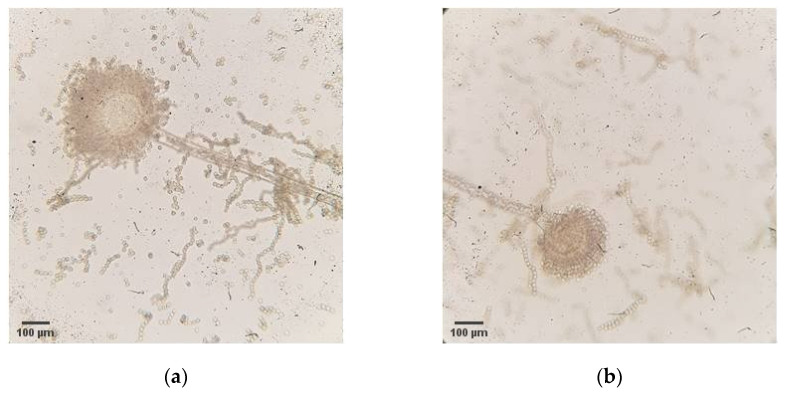

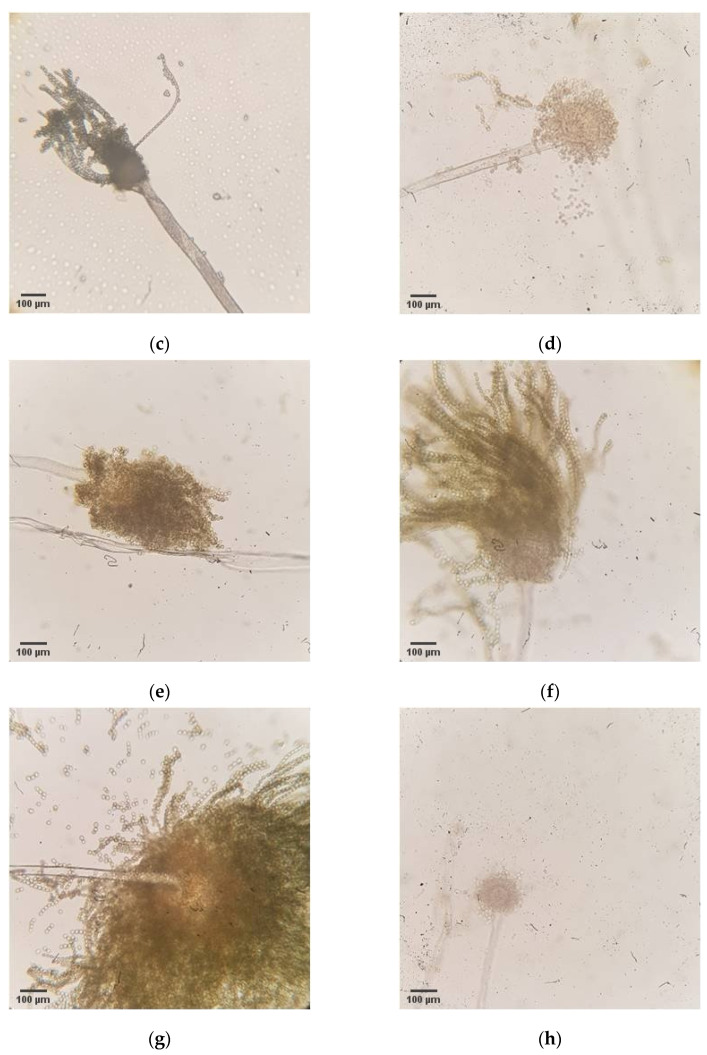

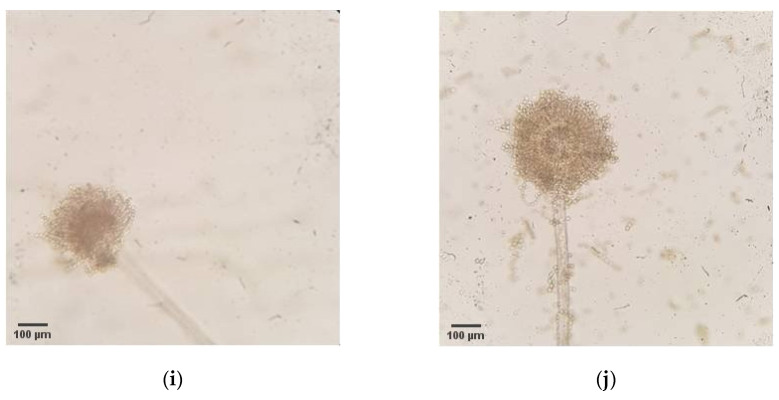
Micromorhological characteristics of *A. flavus* strains. (**a**)—ŠT2BSS, (**b**)—VA1BSS, (**c**)—PA2DSS, (**d**)—SA2BSS, (**e**)—SU1ASS, (**f**)—NA2BSS, (**g**)—LO1ASS, (**h**)—BČ1CSS, (**i**)—SO1ASS, (**j**)—RO2BSS.

The results of microscopic observations of the *Aspergillus* strains are presented in Figure A2 (Appendix D). Micromorphology of the isolated strains indicated the presence of colourless, smooth, or finely roughened thick-walled conidiophores. The conidiophores were unbranched and non-septated. The conidia shape was radial to elliptic, while vesicules were globose to sub-globose. Phialides were loosely packed, radiating in all directions from metulae. Based on the presented morphological characteristics and previous isolation of potential aflatoxin producers using the selective medium, all 10 isolated strains were identified as members of the *A. flavus* species.

### 2.3. CAP Analysis of the Genetic Profiles of A. flavus Strains

**Figure 1:**
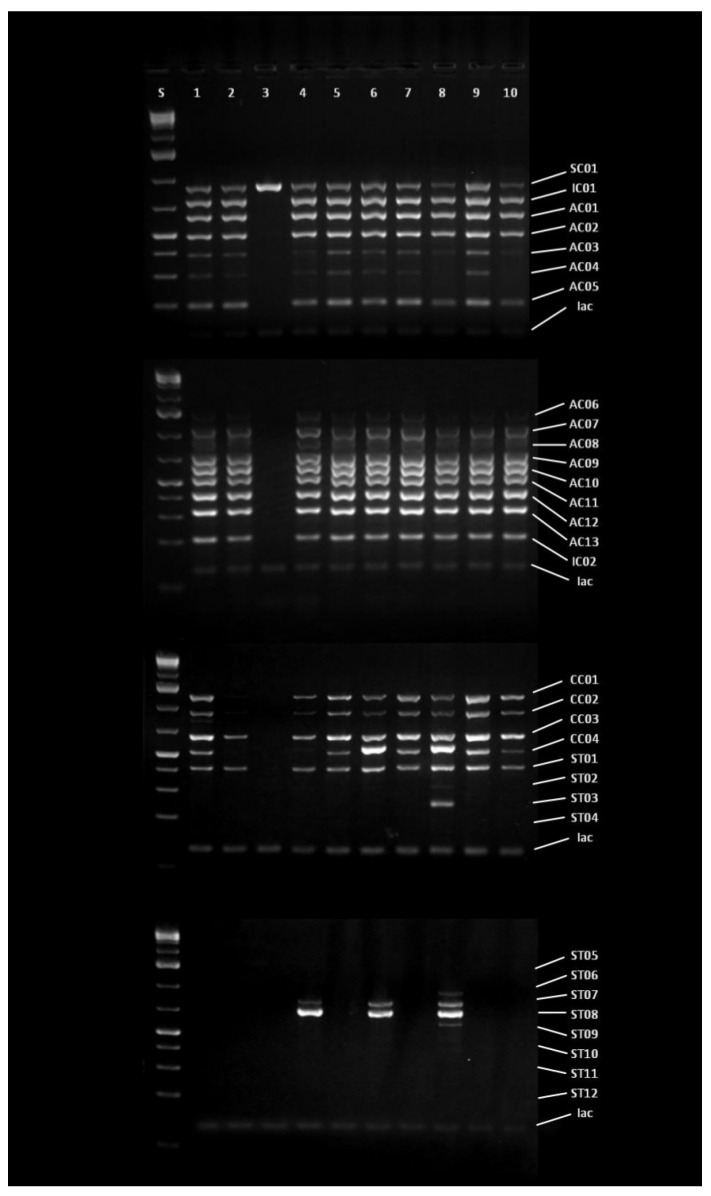
Multiplex PCR amplicons—CAP analysis of the genetic profiles of *A. flavus* isolates. S—GeneRuler 1 kb Plus DNA ladder (Thermo Fischer), 1—VA1BSS, 2—LO1ASS, 3—RO2BSS, 4—BČ1CSS, 5—SO1ASS, 6—SA2BSS, 7—SU1ASS, 8—PA2DSS, 9—ŠT2BSS, 10—NA2BSS. Primers used in multiplex PCR: SC01, IC01, AC01, AC02, AC03, AC04, AC05, AC06, AC07, AC08, AC09, AC10, AC11, AC12, AC13, IC02, Iac, CC01, CC02, CC03, CC04, ST01, ST02, ST03, ST04, ST05, ST06, ST07, ST08, ST09, ST10, ST11, ST12 [28].

Ten monosporial strains of *A. flavus* (further designated as SS—single spore) were selected to determine the aflatoxigenic potential by molecular characterization based on Cluster Amplification Patterns (CAP) analysis. The applied molecular technique considers screening deletions in the aflatoxin biosynthesis gene cluster [11]. A total number of 32 CAP markers spaced approximately every 5 kb along 157 kb of the subtelomere region were amplified in four multiplex PCRs [28]. Figure 1 represents the results of the multiplex PCR analysis for 10 monosporial strains of *A. flavus* isolated from the maize sampled at different localities in the Republic of Serbia in 2019. The obtained results pointed out that nine out of 10 strains show a genetic potential for aflatoxin synthesis. In contrast, only one strain originated from the locality Rogojevac (RO2BSS) possesses significant deletions in the target region, implying atoxigenic character. Making a comparison between nine aflatoxigenic strains, it could be noticed there is a difference in the genetic profile of genes responsible for aflatoxin synthesis among tested strains. The obtained genetic profile of strain from Loznica corresponds to the registered contamination of the maize samples from which it was previously isolated.

### 2.4. Assessment of Aflatoxigenic Potential of A. flavus Strains by Artificial Inoculation

Artificial inoculation of the maize seed samples was performed to assess the aflatoxigenic potential of the *A. flavus* strains characterized as potential aflatoxin producers based on the CAP genetic profiles analysis done in the previous investigation step. The artificial inoculation of the maize seed samples aimed to prove the ability of the selected *A. flavus* strains to produce aflatoxins under simulated favorable environmental conditions, i.e., to confirm expression of the genes responsible for aflatoxin biosynthesis and activation of a corresponding metabolic pathway in the presence of suitable environmental induction factors. After seven days of incubation, the HPLC method was employed to determine aflatoxin presence and content in the infected samples. The results of the HPLC analysis (Table 5) revealed seven samples that tested positive for the presence of aflatoxins: VA1BSS, LO1ASS, SO1ASS, SA2BSS, SU1ASS, PA2DSS, NA2BSS. On the other hand, when it comes to *A. flavus* strains BČ1CSS, ŠT2BSS, and RO2BSS the expression of genes responsible for aflatoxin biosynthesis did not occur, and no aflatoxin contamination was registered in maize inoculated with these strains. The strain RO2BSS was previously characterized as atoxigenic due to detected gene deletions and results obtained after the artificial inoculation confirmed the lack of potential for the aflatoxin synthesis.

### 2.5. Potential Bacillus spp. Antagonistic Strains Isolation 

In the present study, 76 potential producing *Bacillus* spp. presented in Table A1 (Appendix A), were isolated from the rhizosphere soil of different vegetable plants, sampled from localities in the Autonomous Province of Vojvodina, Republic of Serbia. The identification was based on conventional techniques according to Bergey’s manual of determinative bacteriology [29]. 

### 2.6. Screening of the Bacillus spp. Antagonistic Activity against Aflatoxigenic A. flavus Strain SA2BSS

The preliminary screening included evaluation of the antagonistic effect of 76 *Bacillus* spp. strains against one aflatoxigenic *A. flavus* (SA2BSS) strain, which previously showed the potential to produce the largest amount of aflatoxin B1 among the ten tested strains. The cultivation broth samples of 76 *Bacillus* strains, obtained after four days of cultivation, were tested in triplicates using the well diffusion method.

The One-way ANOVA results (Table 6) pointed out the significant effect of the producing strain on the obtained inhibition zone diameters, confirming the variations of the antagonistic activity against the *A. flavus* phytopathogen among the tested *Bacillus* spp. strains (*p* ≤ 0.05).

Mean values and standard deviations of the inhibition zone diameters obtained by testing cultivation broth samples of 76 producing strains against aflatoxigenic *A. flavus* SA2BSS isolate are presented in Table A1 (Appendix B). Duncan’s multiple range test was used to define homogenous groups of producing strains at the same level of statistical significance. The highest inhibitory effect was expressed by strains Mah 1a and Kro 4a, which were at the same level of statistical significance, followed by 23 more strains that showed inhibitory effect against the tested phytopathogen. The remaining 51 strains did not show any antagonistic activity. 

Ten strains with the highest inhibitory activity registered against aflatoxigenic *A. flavus* SA2BSS were selected to investigate broader spectrum antimicrobial activity against a larger number of *A. flavus* strains isolated from corn samples in 2019 from 10 localities in the Republic of Serbia to select an appropriate antagonist for suppression of aflatoxin producers.

### 2.7. Selection of Bacillus Antagonist for Suppression of Aflatoxigenic A. flavus Strains

The following screening step included 10 *Bacillus* spp. with the highest antagonistic potential selected after the preliminary testing of inhibitory activity against an aflatoxigenic strain *A. flavus* SA2BSS. The strains with the highest suppressive effect were tested against strains of *A. flavus* isolated from maize samples harvested at 18 different localities in the Republic of Serbia in 2019). This screening step included both toxigenic and atoxigenic *A. flavus* strains. 

The testing of the cultivation broth samples of 10 *Bacillus* producing strains was performed in the same manner as in the previous investigation step, followed by a similar statistical analysis of the obtained experimental data. The One-way ANOVA results, given in Table 7, again confirmed the significant effect of the producing strain on the obtained inhibition zone diameters, with *p*-value less than 0.05.

**Figure 2:**
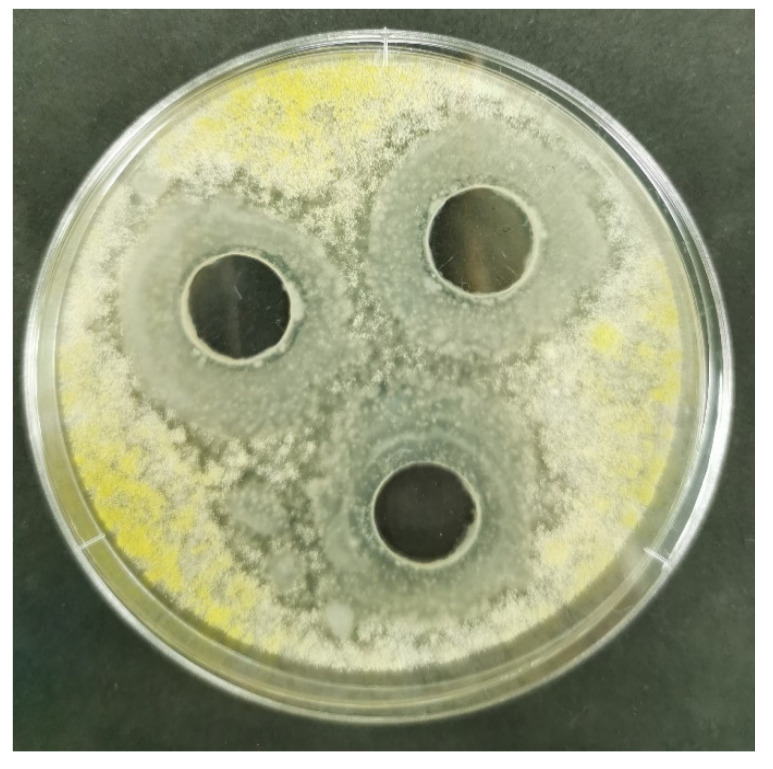
Inhibiton zones obtained using cultivation broth sample of *Bacillus* sp. Mah 1 strain against aflatoxigenic *A. flavus* SA2BSS.

Mean values and standard deviations of the inhibition zone diameters obtained by testing cultivation broth samples of 10 selected *Bacillus* spp. against *A. flavus* strains obtained from 18 different localities in the Republic of Serbia during 2019 are presented in Table 8, grouped using Duncan’s multiple range test in homogenous groups of the same statistical significance. Six out of ten strains expressed antimicrobial activity against all tested *A. flavus* strains. The most intensive suppressive activity was exhibited by the strains designated as Mah 1a (Figure 2) and Mah 1b, which are classified in the group of the same level of statistical significance. 

## 3. Discussion

The sampling of maize harvested in 2019 and 2020 was performed at 18 and 10 different localities in the Republic of Serbia, respectively. The samples were investigated for the presence of aflatoxin B1 using the ELISA test (Table 3 and Table 4), and the content of aflatoxin B1 and total aflatoxins content were determined using the HPLC method (Table 1 and Table 2). The results indicated only one sample with positive result originated from the territory of Loznica, whose total aflatoxin content was below the limit defined by the legislative in the Republic of Serbia (0.002 mg/kg), and the sample was considered as safe from the aspect of food safety. Therefore, based on the presented results, it could be concluded that these two growing seasons resulted in the production of aflatoxin-free or health-safe maize in the selected localities in the Republic of Serbia. In terms of weather conditions, both years were characterized as warm seasons with average annual precipitation rate, and heavy rains during May and June in 2019, and June of 2020 [30,31]. Described weather conditions are defined as convenient for undisturbed maize production and timely harvest [32]. Previous studies indicated the weather conditions influence the incidence and level of aflatoxin contamination of maize grown in the Republic of Serbia. For instance, in the period from 2009–2011 occurrence of aflatoxins in maize samples was not detected [33]. A significantly different scenario happened only a year later. Weather conditions changes, including hot and dry spring and summer in 2012, and drought period that lasted from June to September, resulted in heavy infections of maize by *A. flavus* and, consequently, significant aflatoxin contamination [34]. The contamination level of the maize samples was in the range from 1.01 to even 86.1 µg/kg [33]. Similar weather conditions but with an absence of prolonged drought period occurred in growing season 2013. The occurrence of aflatoxins in maize this season indicated a lower contamination frequency of aflatoxins (24.7%) in comparison to 2012 (72.2%) [35]. In contrast with the weather conditions in 2012 and 2013, in 2014 an extreme amount of precipitation was recorded. The increased moisture created unfavorable conditions for *A. flavus* infections and resulted in absence of aflatoxins in maize samples. A year later, aflatoxin contamination was again recorded (36.5%), but in 2016 high precipitation rate limited growth of the aflatoxigenic fungi (5% of contaminated maize samples) [35]. Vegetation season in 2017 was warmer and dryer above average weather conditions, and results of the analyses for the aflatoxin presence in maize samples collected in the Autonomous Province of Vojvodina, northern agricultural part of the Republic of Serbia, indicated a contamination level of 67% [36]. 

Afterwards, isolation of potential aflatoxin producers was performed using the obtained maize samples from 2019 from 18 locations. The selective medium was used during isolation to target *A. flavus* strains, which show the greatest potential to produce aflatoxins [27]. Macromorphological (Figure A1—Appendix C) and micromorphological (Figure A2—Appendix D) characterization was applied to confirm the belonging of the isolated strains to the species *A. flavus*. Despite originating from different localities, most strains have shown similarities in morphological traits, which correspond to the morphological characteristics specific for the *A. flavus* species [37,38].

The aflatoxigenic potential of the isolated *A. flavus* strains was confirmed by the CAP analyses, previously successfully applied to address the genetic potential for aflatoxins production [15]. The results of CAP analyses have suggested the high distribution of the strains with the genetic potential to produce aflatoxins on the territory of the Republic of Serbia. The results pointed out that even 90% of the strains had a genetic basis for the aflatoxins synthesis, while only one strain isolated from maize sample from the locality Rogojevac (RO2B) had significant deletions in the aflatoxin biosynthesis gene cluster. The inability of this strain to produce aflatoxins is lately confirmed by the artificial inoculation of the maize seeds and the HPLC analyses to determine the content of the produced aflatoxins during artificial inoculation. Methods for monitoring indels within gene clusters required for the biosynthesis of aflatoxins and cyclopiazonic acid (CPA) are used for detecting intraspecies variability of *A. flavus*, but also for the selection of isolates with atoxigenic properties as potential biocontrol agents [28,39,40,41]. Based on deletions and insertions of nucleotides in the sequence of an aflatoxigenic gene, a pattern that implicates stability of toxigenic properties is created. Therefore, cluster amplification pattern (CAP) analysis provides information about the stability of atoxigenic isolates [28], but also for potential and stability in the synthesis of aflatoxins and CPA. The absence of deletions in both aflatoxin and CPA clusters may be a criterion for the selection of toxigenic isolates given that many authors stated additive or even synergistic effects of aflatoxins and CPA [42,43,44]. The aim of this research was to determine the isolates’ capacity for aflatoxin biosynthesis, to select the most stable and potent isolate in aflatoxin production, and to test the efficacy of biocontrol agent based on *Bacillus* spp. against primarily toxigenic isolates. Therefore, genes for aflatoxins and CPA were observed in the first place. Sugar cluster, adjacent to aflatoxin and CPA clusters was also monitored for deletions, however, according to available literature sources, the sugar gene cluster has no direct influence on aflatoxin biosynthesis or the expression on genes in the aflatoxin cluster. Nevertheless, there are data about the possible indirect relationship between these two clusters [45]. Aflatoxin formation relies upon carbon source in a way that simple sugars (glucose, sucrose, fructose, and maltose) support aflatoxin synthesis, while peptone, sorbose, or lactose does not. Additionally, close proximity between the two gene clusters indicates a linkage between them in the metabolism of carbohydrates leading to the induction of aflatoxin biosynthesis. Further, the *nadA* gene in the aflatoxin biosynthetic pathway was considered to be a part of the sugar cluster, however, gene profiling studies using microarray proved that this gene belongs to the aflatoxin gene cluster and has a role in AFG1/AFG2 formation [45,46].

Variations in profiles obtained by the CAP analysis indicate different toxigenic profiles that are in relation to the stability in the biosynthesis of aflatoxin in artificially inoculated samples. Isolates that expressed the highest potential for aflatoxin production (VA1BSS, SA2BSS, PA2DSS) and those with lower detected aflatoxin levels (LO1ASS, SO1ASS, SU1ASS, NA2BSS) have similar profiles. Also, a similar CAP profile in the aflatoxin cluster (AC01-AC13) have isolates with no aflatoxin detected in the maize sample (BČ1CSS, ŠT2BSS). This could be explained by the fact that the synthesis of aflatoxins depends on various factors that can modulate the expression of genes responsible for coding enzymes that control the biosynthesis pathway [10]. These may also include environmental factors which may activate different cell signaling pathways that can affect the expression of the genes involved in toxin production. The inability of aflatoxin production is a result of deletions which are common for the genes involved in the early stages of aflatoxin biosynthesis. In contrast, genes responsible for the later stages are usually characterized by the presence of single nucleotide polymorphisms (SNPs) [47]. Generated genetic profiles of the aflatoxin biosynthesis gene cluster indicated intraspecies variability between the aflatoxigenic strains, which could be classified into four groups. Genetic diversity among *A. flavus* strains isolated from different localities could be explained by the difference in cropping practice employed in a certain field [48]. Additional reasons are gene flow as a result of human activities as well as different competition strategies of *A. flavus* strains depending on the environmental conditions [48]. 

Climate conditions are critical factors for the growth and development of *A.flavus* and subsequent aflatoxin biosynthesis [15]. The 2019 and 2020 seasons in the Republic of Serbia were similar and unfavorable for the development of the *A. flavus* in terms of weather conditions. The results of this study confirmed the lack of maize contamination, except for one sample (from the locality Loznica from 2019), but with the aflatoxin content below the permissible limit. However, proven genetic potential and confirmed gene expression resulting in the high amount of produced AFB1 and total aflatoxins after artificial inoculation of maize seeds have demonstrated the remarkable capacity of the *A. flavus* strains present in the fields of the Republic of Serbia to produce aflatoxins. Favorable climate conditions, drought, and heat stress increase the probability of *A. flavus* development and pose a risk for a high level of aflatoxin contamination in maize as an entry point of a food chain. This kind of scenario combined with unpredictable consequences of climate changes implies the necessity of strict control of *A. flavus* distribution in the fields. Considering the maize as a crop of strategic importance in the Republic of Serbia, the consequences of the potential damage due to the outbreak of aflatoxin contamination would dramatically influence the country’s economy and food and feed safety, as previously seen in season 2012 [49]. If climate conditions changes initiate more regular aflatoxin contamination in the United States of America, as the largest maize producer, it was estimated that losses to the maize industry could reach from $52.1 million to $1.86 billion annually [50]. Therefore, this study was also focused on the investigation of sustainable biocontrol methods for suppression of *A. flavus*, as a means of preventive action for aflatoxin contamination emergence. On the other hand, the production of aflatoxins brings the more severe consequence of *A. flavus* presence in the maize fields, but what also should be taken into account are economic losses due to maize fungal infection. Fungal diseases of cereals can cause a yield reduction in the range from 15–20%, but even more in some extreme cases (60%) [51]. 

Screening of the bacteria belonging to the *Bacillus* genus as a promising biocontrol agent revealed intensive suppressive activity exhibited by the Mah 1a and Mah 1b strains against toxigenic and atoxigenic *A. flavus* strains, isolated from the maize grown in the Republic of Serbia. The preliminary screening included 76 *Bacillus* strains as potential antagonists against one aflatoxigenic *A. flavus* (SA2BSS) strain, with the highest potential of aflatoxin B1 production. On the other hand, the main screening experiment included all isolated *A. flavus* strains to evaluate if there is a difference in the activity of *Bacillus strains* on *A. flavus* population. Both antagonistic strains characterized by the highest inhibitory activity (Mah1a and Mah1b) were isolated from the rhizosphere soil of the *Phaseolus vulgaris*. Additionally, antagonistic and phytopathogenic strains originate from the same region, which contributes to the efficiency and adaptation capability of selected biocontrol agents to the environmental conditions of the potential application site [21]. The rhizosphere is a great source of beneficial bacterial strains, and almost 95% of the soil Gram-positive bacilli are a member of the *Bacillus* genus [52]. The *Bacillus* strains isolated from the rhizosphere soil of the *Phaseolus vulgaris* were previously defined as strains of agricultural interest due to their plant-beneficial and pathogen-suppressing activities [22,53,54]. Production of extracellular enzymes is of great importance since it contributes to biocontrol activity and adaptation to the environmental conditions, giving those strains a more competitive advantage over the other microbial inhabitants of the particular ecosystem [52]. The *Bacillus* strains are marked out as core members of the microbiome in *Phaseolus vulgaris* rhizosphere [55]. Moreover, *Bacillus* spp. Isolated from the *Phaseolus vulgaris* rhizosphere stood out by their superiority among other strains thanks to their plant growth promotion characteristics and potential for antimicrobial metabolites production [56]. Hence, the isolated *Bacillus* strains Mah 1a and Mah 1b successfully inhibited the growth and development of *A. flavus* in vitro. These strains are currently being further investigated as potential biocontrol agents for the suppression of fungal maize diseases and aflatoxin contamination. Previous studies also indicated the use of *Bacillus* spp. For the suppression of fungal pathogens [57,58,59,60], including *Aspergillus* species [18,61,62,63]. Further research from the aspect of biocontrol product development should first include identifying the selected antagonists and the precise determination of the mechanism of antifungal activity. Depending on the previously defined mechanism of action, further steps in bioprocess development should be determined to achieve the maximization of microbial biomass or metabolites production. Development of the bioprocess solution should include optimization of the medium composition, bioprocess parameters, and downstream procedure for the production of the microbial biocontrol agent. The initial investigation steps should be performed at the laboratory scale with the perspective of scaling up the developed production technology to pilot or industrial level [21,64,65]. All these phases should be followed by *in planta* testing under field conditions to obtain a highly efficient biocontrol product for suppression of *A. flavus* and an eco-friendly tool for preventive action against aflatoxin contamination outbreaks.

## 4. Materials and Methods

### 4.1. Isolation of Fungal Strains

All *A*. *flavus* strains examined in this study were isolated from maize sampled during the 2019 harvest season. Collected maize samples from 18 localities (Pančevo, Užice, Loznica, Subotica, Valjevo, Sirig, Novi Sad, Bečej, Sombor, Maglić, Karavukovo, Nadalj, Kulpin, Sivac, Sabanta, Štitar, Lepojević, Rogojevac) were ground, placed in sterile paper bags and stored at 4 °C until use. Ground maize aliquots of 5 g each were suspended in 25 mL of sterile water and spread on the selective isolation medium—Clean up (CU) medium, supplemented with 5 mg/mL of Bengal Rose and 1 mg/mL of Dichloran, and amended with the antibiotics (10 mg/mL of Chloramphenicol and 10mg/mL Streptomycin) [66]. Isolations were performed in three replicates per sample and CU plates were incubated at 31 °C for three days. Three-day-old plates were examined, and the total number of *A*. *flavus* colonies were recorded. Plates with ten or fewer colonies were selected, and pick-ups were conducted by lightly touching one conidiophore of the discrete *A. flavus* colony and by single point inoculation of the center of 5–2 medium (5% V-8 juice, 2% agar, pH 5.2, 1000 mL H_2_O) [27]. Plates were incubated for 7–10 days at 31 °C. *Aspergillus* section *Flavi* strains were identified to the species level based on macroscopic and microscopic morphological characteristics [27]. Pure cultures were stored in sterile water (six colonized agar plugs with a diameter of 3mm added to 1.5 mL of sterile water) and deposited at the Microbial culture collection of Laboratory for detection of pathogens, pests, and weeds of Faculty of Agriculture, University of Novi Sad.

### 4.2. Selection of A. flavus Strains and Single Spore Isolation

For further analysis and accurate identification, phenotypic purity of *A. flavus* strains was achieved through single-spore culturing. Single spore isolations of selected strains were conducted by seral dilutions of spore suspension of strains cultivated at 31 °C for 7–10 days on 5-2 agar medium—100 µL of the most diluted suspensions (10^−5^ to 10^−8^ dilutions) were transferred to 1% Malt agar (HiMedia Laboratories, Mumbai, India) medium and incubated at 31 °C for 24–48 h. Plates with ten or fewer colonies were selected and single colonies were transferred to the center of 5-2 medium at 31 °C for 7–10 days. Single spore strains were stored in sterile water and deposited at Microbial culture collection of Laboratory for detection of pathogens, pests, and weeds of Faculty of Agriculture, University of Novi Sad, Novi Sad, Republic of Serbia.

### 4.3. DNA Extraction from the A. flavus Strains

Ten *A. flavus* single spore strains characterized based on the colony and spore morphology were selected for further study. To prepare DNA, *A. flavus* strains were center-point inoculated onto 5-2 agar medium and incubated 8–10 days at 31 °C. The total genomic DNA was extracted using Cetyltrimethyl Ammonium Bromide (CTAB) method [67]. Purified DNA was used as a template for the PCR (polymerase chain reaction) amplification for Cluster Amplification Patterns (CAP) analysis.

### 4.4. Monitoring Deletions in the Aflatoxin Biosynthesis Gene Cluster of Selected A. flavus Strains

Cluster amplification pattern markers were amplified in four multiplex PCRs [28]. PCR was performed in 10 μL volumes using 6 ng of genomic DNA, 0.08 µmol of each primer and SuperHot MasterMix 2x (Bioron, Römerberg, Germany) on Surecycler 8800 Thermal Cycler (Agilent Technologies, Santa Clara, CA, USA) under the following conditions: 94 °C for 1 min, followed by 30 cycles at 94 °C for 30 s, 62 °C for 90 s, 72 °C for 90 s and the final extension step of 72 °C for 10 min. Products were visualized on 1.4% agarose gel in 1 × sodium boric acid buffer [68].

### 4.5. Quantitative Determination of Aflatoxin B1 and Total Aflatoxins 

#### 4.5.1. ELISA Analysis of Aflatoxin B1 Presence in Maize Samples

Determination of Aflatoxin B1 content in maize samples collected at different locations in the Republic of Serbia (Table 3 and Table 4) in 2019 and 2020 was performed using AgraQuant^®^ Aflatoxin B1 ELISA Test Kit (Romer Labs GmbH, Tulln an der Donau, Austria). Each ground maize sample (20 g) was extracted with 100 mL of 70% methanol and vigorously shook using Benchtop Shaking Incubator 222DS (Labnet International Inc., Edison, NJ, USA) at 200 rpm for 5 min. The sample was allowed to settle, and the top layer of the extract was filtered through a Whatman #1 filter paper (Whatman, UK). Afterwards, 100 µL of the collected filtrate was diluted using the assay buffer. To perform the analysis, 100 µL of each sample or standard was mixed with 200 µL of the conjugate in individual dilution wells, and then 100 µL from each dilution well was transferred to a respective antibody-coated microwell. After 15 min of incubation at room temperature, the plate was washed with distilled water and 100 µL of substrate solution was added to each well, allowed to incubate for 5 min, and then stop solution (100 µL for each well) was added. The absorbance of each well was read at 450 nm (reference wavelength 630 nm) within 10 min after the addition of stop solution using the Microplate Photometer HiPo MPP-96 (BioSan, Rīga, Latvia).

#### 4.5.2. HPLC Quantitative Analysis of Aflatoxins’ Content in Maize Samples

Chromatographic determination of aflatoxins was carried out on a 1260 series HPLC system (Agilent Technologies, Santa Clara, CA, USA) with a DAD (Diode-Array Detector) and FLD (Fluorescence Detector) detectors (Agilent Technologies, USA) and a Hypersil ODS (150 × 4.6 mm i.d., particle size 5 μm) column (Agilent Technologies, USA). Exactly 12.5 g of samples were extracted using 50 mL of acetonitrile (St. Louis, MO, United States) and water mixture (84:16, *v*/*v*). The extracts were then cleaned up on MycosepTM 224 column (Romer Labs. Inc., Union, MO, USA). Thereafter, 3 mL of cleaned-up extract was evaporated just to dryness at 60 °C under gentle steam of nitrogen. The residue was dissolved in 300 µL of the mobile phase. HPLC conditions were determined according to Oliveira et al. (2009) [69]. All analyses were conducted in duplicate.

### 4.6. Potential for Aflatoxin Production in Maize

Artificial inoculation of healthy, uncontaminated (previously analysed by HPLC and ELISA) and undamaged, sterile maize seeds was carried out according to the method described by Probst and Cotty [70] with a few modifications, using the *A. flavus* strains isolated from maize samples collected during 2019 (ŠT2BSS, VA1BS, PA2DSS, SA2BSS, SU1ASS, NA2BSS, LO1ASS, BČ1CSS, SO1ASS, RO2BSS). Maize seeds (100 g) were placed in 1 L glass jars with perforated lids. Grain moisture was adjusted to 25% moisture content by adding sterile water. Grains were periodically shaken to achieve uniform moisture distribution. Thereafter, grains were autoclaved for 20 min at 121 °C and 2.1 bar. For the inoculum preparation, conidia from 6-day old cultures of *A. flavus* strains were suspended in sterile distilled water, adjusted to a concentration of 10^5^ spore/mL, and added to each glass jar. The inoculated seeds were incubated for 5–7 days at 30 °C and analysed for aflatoxin contamination (total aflatoxin, aflatoxin B1) by HPLC (as previously described in Section 4.5.2.). Aflatoxin formation is directly affected by temperature. Optimal aflatoxin production is observed at temperatures near 30 °C (28 °C to 35 °C), hence this temperature was selected for the assay [45]. Uninoculated seeds of maize harvested in 2020 were used as control samples and were previously characterized as non-contaminated by aflatoxins using the HPLC method and ELISA test (Section 2.1). 

### 4.7. Isolation of Antagonists

A total number of 76 potential producing strains (antagonists against *A. flavus*) was isolated from the rhizosphere soil, sampled from different localities in the Autonomous Province of Vojvodina, Republic of Serbia. The selective isolation of sporogenic strains was performed by resuspending 1 g of rhizosphere soil samples in 9 mL of saline and incubating at 28 °C for 15 min at 150 rpm. After homogenisation, thermal treatment at 100 °C for 7 min was performed. Dilution series (10^−1^, 10^−2^, 10^−3^) were prepared, and 500 µL of each dilution was inoculated on nutrient agar (Himedia Laboratories, Mumbai, India) plates and incubated at 28 °C for 48 h. The next step included obtaining pure cultures of morphologically different strains, which were selected according to their morphological and biochemical traits [29] indicating belonging to the *Bacillus* genus. The selected colonies were picked by a sterile loop and inoculated on nutrient agar plates, followed by incubation at 28 °C for 48 h. The isolated pure cultures were stored on nutrient agar slant at 4 °C at the culture collection of the Laboratory for Biochemical Engineering, Faculty of Technology Novi Sad, University of Novi Sad.

### 4.8. Inoculum Preparation and Cultivation of Bacillus spp. Antagonists

The inoculum preparation started by incubation of the potential producing *Bacillus* spp. strains on nutrient agar for 48 h at 28 °C. The second step included transferring the loopful biomass of antagonists into the liquid media (nutrient broth—Himedia, Laboratories, Mumbai, India) and incubating at 28 °C for 48 h on a rotary shaker with an agitation rate of 170 rpm. Inoculation of cultivation media was performed by adding 10% (*v*/*v*) of the prepared inocula (5 mL) to the Erlenmeyer flasks containing 50 mL of nutrient broth (Himedia Laboratories, Mumbai, India). The cultivation was carried out on a rotary shaker for 96 h, with a temperature of 28 °C, with an agitation rate of 170 rpm.

### 4.9. Antimicrobial Activity Assay

The biomass of phytopathogenic *A. flavus* strains was suspended in sterile saline to achieve a spores’ concentration of 10^5^ CFU/mL. Sabourad maltose agar media (Himedia Laboratories, Mumbai, India) were melted and tempered (50 ± 1 °C) and, before pouring into the Petri dishes, inoculated using 1 mL of the prepared suspensions. The well diffusion method in triplicate tests was employed to evaluate the antagonistic effect of the cultivation broth samples (100 µL) obtained after 96 h of cultivation of the selected *Bacillus* sp. antagonists against the tested phytopathogens. The incubation was performed at 30 °C for 96 h, and followed by the inhibition zone diameters measurement.

### 4.10. Statistical Analysis

The analysis of the obtained experimental results included calculating the average values and standard deviations of the measured inhibition zone diameters using Microsoft^®^ Excel 2010 software (Microsoft Corporation, DC, USA). Statistical data analyses were performed using Statistica 13.5 software (Tibco Software Inc., Carslbad, CA, USA), and the employed methods were ANOVA and post hoc testing using Duncan’s multiple range test. All statistical analyses were performed at the significance level of 0.05.

## Figures and Tables

**Table 1:** Aflatoxins content determination in maize samples collected from 10 different localities in the Republic of Serbia in 2019 by the HPLC method.

Locality	Strain Mark	AF B1 (mg/kg)	Total (mg/kg)
Loznica	LO	0.002	0.002
Sombor	SO	<0.001	<0.001
Subotica	SU	<0.001	<0.001
Pančevo	PA	<0.001	<0.001
Bečej	BČ	<0.001	<0.001
Sabanta	SI	<0.001	<0.001
Nadalj	NA	<0.001	<0.001
Valjevo	VA	<0.001	<0.001
Rogojevac	RO	<0.001	<0.001
Štitar	ŠT	<0.001	<0.001

**Table 2:** Aflatoxins content determination in maize samples collected from 10 different localities in the Republic of Serbia in 2020 by the HPLC method.

Locality	Strain Mark	AF B1 (mg/kg)	Total (mg/kg)
Rumenka	RU	<0.001	<0.001
Oparić	OP	<0.001	<0.001
Kuzmin	KU	<0.001	<0.001
Lepojević	LE	<0.001	<0.001
Martinci	MC	<0.001	<0.001
Krušedol	KŠ	<0.001	<0.001
Valjevo	VA	<0.001	<0.001
Beška	BŠ	<0.001	<0.001
Bečej	BČ	<0.001	<0.001
Sombor	SO	<0.001	<0.001

**Table 3:** Aflatoxin B1 presence determination in maize samples harvested in 2019 collected from 10 different localities in the Republic of Serbia by the ELISA test.

Locality	Strain Mark	AF B1 (mg/kg)
Štitar	ŠT	0.0000
Valjevo	VA	0.0000
Pančevo	PA	0.0000
Sabanta	SI	0.0000
Subotica	SU	0.0000
Vršac	NA	0.0000
Loznica	LO	0.0046
Bečej	BČ	0.0000
Sombor	SO	0.0000
Rogojevac	RO	0.0000

**Table 4:** Aflatoxin B1 presence determination in maize samples harvested in 2020 collected from 10 different localities in the Republic of Serbia by the ELISA test.

Locality	Strain Mark	AF B1 (mg/kg)
Rumenka	RU	0.0000
Oparić	OP	0.0000
Kuzmin	KU	0.0000
Lepojević	LE	0.0000
Martinci	MC	0.0000
Krušedol	KŠ	0.0000
Valjevo	VA	0.0000
Beška	BŠ	0.0000
Bečej	BČ	0.0000
Sombor	SO	0.0000

**Table 5:** Aflatoxins content determination in artificially inoculated maize seed samples by the HPLC method as assessment of aflatoxigenic potential of 10 *A. flavus* strains.

Locality of Strain Origin	Strain Used for Inoculation	AF B1 (mg/kg)	Total AF (mg/kg)
Štitar	ŠT2BSS	<0.001	<0.001
Valjevo	VA1BSS	989.4	2217.6
Pančevo	PA2DSS	1281.3	1891.0
Sabanta	SA2BSS	1354.4	2147.0
Subotica	SU1ASS	445.7	838.8
Nadalj	NA2BSS	102.7	321.8
Loznica	LO1ASS	347.9	962.4
Bečej	BČ1CSS	<0.001	<0.001
Sombor	SO1ASS	330.4	564.6
Rogojevac	RO2BSS	<0.001	<0.001
Uninoculated control		<0.001	<0.001

**Table 6:** One-way ANOVA of inhibition zone diameters for cultivation broth samples of *Bacillus* spp. antagonists used for suppression of aflatoxigenic *A. flavus* SA2BSS.

Effect	SS	DF	MS	F-value	*p*-Value
Intercept	13,678.75	1	13,678.75	5142.219	0.00
Antagonist	31,264.41	75	416.86	156.709	0.00
Error	404.33	152	2.66		

SS—sum of squares, MS—mean squares, DF—degree of freedom.

**Table 7:** One-way ANOVA of inhibition zone diameters for cultivation broth samples of 10 selected *Bacillus* spp. antagonists used for suppression of toxigenic and atoxigenic *A. flavus* strains.

Effect	SS	DF	MS	F-Value	*p*-Value
Intercept	174,074.1	1	174,074.1	1422.828	0.00
Antagonist	200,611.7	9	22,290.2	182.193	0.00
Error	123,567.2	1010	122.3		

SS—sum of squares, MS—mean squares, DF—degree of freedom.

**Table 8:** Duncan’s multiple range test results—mean values and standard deviations of inhibition zone diameters obtained using cultivation broth samples of 10 selected *Bacillus* spp. against toxigenic and atoxigenic *A. flavus* strains.

Antagonist	Inhibition Zone Diameter (mm)
Šar 3b	0.00 ± 0.00 ^a^
Šar 1	0.00 ± 0.00 ^a^
Pap 2a	0.00 ± 0.00 ^a^
Pap 3	0.00 ± 0.00 ^a^
Paš 1b	7.97 ± 12.75 ^b^
Par 3	11.27 ± 16.74 ^c^
Šar 3a	14.74 ± 16.50 ^d^
Kro 4a	22.18 ± 18.49 ^e^
Mah 1b	36.96 ± 9.81 ^f^
Mah 1a	37.52 ± 8.82 ^f^

Superscript letters ^(a–f)^ represent different levels of statistical significance. Values marked with the same letter are at the same level of significance.

## Data Availability

Not applicable.

## Appendix A

**Table A1:** Potential antagonists isolated from the rhizosphere soil of different vegetable plants originated from localities in the Autonomous Province of Vojvodina, Republic of Serbia.

*Bacillus* sp. Potential Antagonist	Rhizosphere Soil Samples Used for Isolation	Locality of Strain Origin
Cve 2b	*Beta vulgaris* subsp. *vulgaris*	Selenča
Luk 1b	*Allium cepa*	Maglić
Luk 2	*Allium cepa*	Maglić
Luk 3	*Allium cepa*	Maglić
Luk 4a	*Allium cepa*	Maglić
Luk 4b	*Allium cepa*	Maglić
Kra 3	*Cucumis sativus*	Bač
Pas 1b	*Phaseolus vulgaris*	Obrovac
Pas 2a	*Phaseolus vulgaris*	Obrovac
Pas 2b	*Phaseolus vulgaris*	Obrovac
Pas 3a	*Phaseolus vulgaris*	Obrovac
Kra 2c	*Cucumis sativus*	Bač
Pas 4a	*Pastinaca sativa*	Obrovac
Pas 4b	*Pastinaca sativa*	Obrovac
Gra 1	*Pisum sativum*	Bačko Novo Selo
Gra 2a	*Pisum sativum*	Bačko Novo Selo
Gra 2b	*Pisum sativum*	Bačko Novo Selo
Gra 3a	*Pisum sativum*	Bačko Novo Selo
Gra 3b	*Pisum sativum*	Bačko Novo Selo
Gra 4a	*Pisum sativum*	Bačko Novo Selo
Gra 4b	*Pisum sativum*	Bačko Novo Selo
Kra 2b	*Cucumis sativus*	Bač
Kra 2a	*Cucumis sativus*	Bač
Mah 2a	*Phaseolus vulgaris*	Tovariševo
Mah 2b	*Phaseolus vulgaris*	Tovariševo
Mah 3a	*Phaseolus vulgaris*	Tovariševo
Mah 3b	*Phaseolus vulgaris*	Tovariševo
Paš 3	*Pastinaca sativa*	Gložan
Mah 4a	*Phaseolus vulgaris*	Tovariševo
Mah 4b	*Phaseolus vulgaris*	Tovariševo
Paš 1a	*Pastinaca sativa*	Gložan
Šar 2a	*Daucus carota* subsp. *sativus*	Begeč
Pap 4b	*Capsicum annuum*	Odžaci
Pap 4a	*Capsicum annuum*	Odžaci
Pap 2b	*Capsicum annuum*	Odžaci
Pap 1b	*Capsicum annuum*	Odžaci
Cve 1	*Beta vulgaris* subsp. *vulgaris*	Selenča
Cve 2a	*Beta vulgaris* subsp. *vulgaris*	Selenča
Kra 4	*Cucumis sativus*	Bač
Cve 3	*Beta vulgaris* subsp. *vulgaris*	Selenča
Cve 4	*Beta vulgaris* subsp. *vulgaris*	Selenča
Kro 1a	*Solanum tuberosum*	Gajdobra
Kro 1b	*Solanum tuberosum*	Gajdobra
Kro 2	*Solanum tuberosum*	Gajdobra
Pap 1a	*Capsicum annuum*	Odžaci
Par 4	*Solanum lycopersicum*	Deronje
Kup 3b	*Brassica oleracea var. capitata*	Futog
Kro 4b	*Solanum tuberosum*	Gajdobra
Kup 1	*Brassica oleracea var. capitata*	Futog
Kup 2	*Brassica oleracea var. capitata*	Futog
Kup 3a	*Brassica oleracea var. capitata*	Futog
Par 1	*Solanum lycopersicum*	Deronje
Kra 1a	*Cucumis sativus*	Bač
Šar 2b	*Daucus carota* subsp. *sativus*	Begeč
Kra 1b	*Cucumis sativus*	Bač
Kro 3a	*Solanum tuberosum*	Gajdobra
Par 2	*Solanum lycopersicum*	Deronje
Paš 2	*Pastinaca sativa*	Gložan
Paš 4	*Pastinaca sativa*	Gložan
Mah 3c	*Phaseolus vulgaris*	Tovariševo
Luk 1a	*Allium cepa*	Maglić
Pas 1a	*Phaseolus vulgaris*	Obrovac
Kro 3b	*Solanum tuberosum*	Gajdobra
Pas 3b	*Phaseolus vulgaris*	Obrovac
Kup 4	*Brassica oleracea var. capitata*	Futog
Par 3	*Solanum lycopersicum*	Deronje
Šar 4	*Daucus carota* subsp. *sativus*	Begeč
Paš 1b	*Pastinaca sativa*	Gložan
Pap 3	*Capsicum annuum*	Odžaci
Šar 3b	*Daucus carota* subsp. *sativus*	Begeč
Šar 3a	*Daucus carota* subsp. *sativus*	Begeč
Pap 2a	*Capsicum annuum*	Odžaci
Mah 1b	*Phaseolus vulgaris*	Tovariševo
Šar 1	*Daucus carota* subsp. *sativus*	Begeč
Mah 1a	*Phaseolus vulgaris*	Tovariševo
Kro 4a	*Solanum tuberosum*	Gajdobra

## Appendix B

**Table A2:** Duncan’s multiple range test results—mean values and standard deviations of inhibition zone diameters obtained using cultivation broth samples of 76 *Bacillus* strains against *A. flavus* SA2BSS.

*Bacillus* sp. Antagonist	Inhibition Zone Dimeter (mm)
Cve 2b	0.00 ± 0.00 ^a^
Luk 1b	0.00 ± 0.00 ^a^
Luk 2	0.00 ± 0.00 ^a^
Luk 3	0.00 ± 0.00 ^a^
Luk 4a	0.00 ± 0.00 ^a^
Luk 4b	0.00 ± 0.00 ^a^
Kra 3	0.00 ± 0.00 ^a^
Pas 1b	0.00 ± 0.00 ^a^
Pas 2a	0.00 ± 0.00 ^a^
Pas 2b	0.00 ± 0.00 ^a^
Pas 3a	0.00 ± 0.00 ^a^
Kra 2c	0.00 ± 0.00 ^a^
Pas 4a	0.00 ± 0.00 ^a^
Pas 4b	0.00 ± 0.00 ^a^
Gra 1	0.00 ± 0.00 ^a^
Gra 2a	0.00 ± 0.00 ^a^
Gra 2b	0.00 ± 0.00 ^a^
Gra 3a	0.00 ± 0.00 ^a^
Gra 3b	0.00 ± 0.00 ^a^
Gra 4a	0.00 ± 0.00 ^a^
Gra 4b	0.00 ± 0.00 ^a^
Kra 2b	0.00 ± 0.00 ^a^
Kra 2a	0.00 ± 0.00 ^a^
Mah 2a	0.00 ± 0.00 ^a^
Mah 2b	0.00 ± 0.00 ^a^
Mah 3a	0.00 ± 0.00 ^a^
Mah 3b	0.00 ± 0.00 ^a^
Paš 3	0.00 ± 0.00 ^a^
Mah 4a	0.00 ± 0.00 ^a^
Mah 4b	0.00 ± 0.00 ^a^
Paš 1a	0.00 ± 0.00 ^a^
Šar 2a	0.00 ± 0.00 ^a^
Pap 4b	0.00 ± 0.00 ^a^
Pap 4a	0.00 ± 0.00 ^a^
Pap 2b	0.00 ± 0.00 ^a^
Pap 1b	0.00 ± 0.00 ^a^
Cve 1	0.00 ± 0.00 ^a^
Cve 2a	0.00 ± 0.00 ^a^
Kra 4	0.00 ± 0.00 ^a^
Cve 3	0.00 ± 0.00 ^a^
Cve 4	0.00 ± 0.00 ^a^
Kro 1a	0.00 ± 0.00 ^a^
Kro 1b	0.00 ± 0.00 ^a^
Kro 2	0.00 ± 0.00 ^a^
Pap 1a	0.00 ± 0.00 ^a^
Par 4	0.00 ± 0.00 ^a^
Kup 3b	0.00 ± 0.00 ^a^
Kro 4b	0.00 ± 0.00 ^a^
Kup 1	0.00 ± 0.00 ^a^
Kup 2	0.00 ± 0.00 ^a^
Kup 3a	0.00 ± 0.00 ^a^
Par 1	17.00 ± 0.00 ^b^
Kra 1a	17.33 ± 1.52 ^bc^
Šar 2b	18.00 ± 1.00 ^bcd^
Kra 1b	18.33 ± 0.57 ^bcde^
Kro 3a	19.00 ± 0.00 ^bcde^
Par 2	19.00 ± 1.73 ^bcde^
Paš 2	19.67 ± 0.57 ^bcdef^
Paš 4	19.67 ± 0.57 ^bcdef^
Mah 3c	20.00 ± 0.00 ^bcdefg^
Luk 1a	20.00 ± 1.00 ^bcdefg^
Pas 1a	20.17 ± 0.76 ^cdefg^
Kro 3b	20.33 ± 0.57 ^cdefg^
Pas 3b	20.67 ± 0.76 ^defg^
Kup 4	20.83 ± 1.15 ^defg^
Par 3	21.33 ± 2.30 ^efgh^
Šar 4	22.67 ± 0.57 ^fghi^
Paš 1b	23.00 ± 1.00 ^ghi^
Pap 3	24.00 ± 1.00 ^hi^
Šar 3b	25.33 ± 4.50 ^i^
Šar 3a	25.33 ± 7.81 ^i^
Pap 2a	29.67 ± 2.51 ^j^
Mah 1b	30.00 ± 5.00 ^j^
Šar 1	35.00 ± 4.50 ^k^
Mah 1a	40.00 ± 0.00 ^l^
Kro 4a	42.33 ± 6.80 ^l^

Superscript letters ^(a–l)^ represent different levels of statistical significance. Values marked with the same letter are at the same level of significance.
